# Immunological Responses and Actin Dynamics in Macrophages Are Controlled by N-Cofilin but Are Independent from ADF

**DOI:** 10.1371/journal.pone.0036034

**Published:** 2012-04-27

**Authors:** Friederike Jönsson, Christine B. Gurniak, Bernhard Fleischer, Gregor Kirfel, Walter Witke

**Affiliations:** 1 Institute of Genetics, Cell Motility Unit, Rheinische Friedrich-Wilhelms-Universität, Bonn, Germany; 2 Department of Immunology, Bernhard-Nocht-Institute for Tropical Medicine, Hamburg, Germany; 3 Département d'Immunologie, Unité d'Allergologie Moléculaire et Cellulaire, Institut Pasteur, Inserm U.760, Paris, France; 4 Institute for Cell Biology, Rheinische Friedrich-Wilhelms-Universität, Bonn, Germany; George Mason University, United States of America

## Abstract

Dynamic changes in the actin cytoskeleton are essential for immune cell function and a number of immune deficiencies have been linked to mutations, which disturb the actin cytoskeleton. In macrophages and dendritic cells, actin remodelling is critical for motility, phagocytosis and antigen presentation, however the actin binding proteins, which control antigen presentation have been poorly characterized. Here we dissect the specific roles of the family of ADF/cofilin F-actin depolymerizing factors in macrophages and in local immune responses.

Macrophage migration, cell polarization and antigen presentation to T-cells require n-cofilin mediated F-actin remodelling. Using a conditional mouse model, we show that n-cofilin also controls MHC class II-dependent antigen presentation. Other cellular processes such as phagocytosis and antigen processing were found to be independent of n-cofilin. Our data identify n-cofilin as a novel regulator of antigen presentation, while ADF on the other hand is dispensable for macrophage motility and antigen presentation.

## Introduction

The actin cytoskeleton controls cellular processes that are important for the efficacy of immune responses. These processes include cell motility [1], endocytosis [2], cell polarity and intracellular trafficking [3]. In macrophages and dendritic cells the actin cytoskeleton has been shown to regulate chemotaxis [4], phagocytosis [5] and antigen presentation [6]. In addition, receptor clustering [7] and T-cell activation [6], [8] have been shown to depend on remodelling of the actin cytoskeleton.

Mutations, which affect cytoskeletal dynamics, can result in severe immunodeficiencies [9]. The Wiskott-Aldrich syndrome for example is caused by a mutation of the actin binding protein WASP [10], leading to defects in migration and chemotaxis of myeloid cells [11]. Certain pathogenic bacteria such as *Listeria* and *Salmonella* exploit the actin cytoskeleton to escape immune responses [12], and the entry of HI-virus to T-cells has been shown to depend on n-cofilin [13].

Macrophages and dendritic cells share many common features and are crucial for the induction of adaptive immune responses as well as first line pathogen defence. They are unique in their ability to infiltrate infected tissues, where they ingest large amounts of pathogens. Upon proteolytic degradation of these pathogens, macrophages and dendritic cells can both present antigenic peptides to lymphocytes [14]. Dendritic cells are specialized antigen presenting cells that migrate from the periphery to the lymph nodes upon encounter of an antigenic stimulus.

Cell motility is controlled by actin binding proteins, which regulate the turnover of actin filaments. The F-actin depolymerizing factors ADF/cofilin are thus likely candidates to modulate immune responses in macrophages and dendritic cells. The family of F-actin depolymerizing factors comprise evolutionary conserved proteins [15], [16], which have a fundamental role in regulating actin filament turnover [17], [18]. In human and mouse, three ADF/cofilin family members can be found – n-cofilin or non-muscle cofilin [19], m-cofilin or muscle cofilin [20] and ADF [21]. For human ADF, also the name ‘Destrin’ is frequently used. ADF/cofilin proteins were shown to control chemotaxis [22], as well as neuronal crest cell migration in the developing embryo [23], [24].

To dissect the functions of the individual actin depolymerizing factor family members in immune responses, we specifically deleted n-cofilin, and ADF in the macrophage lineage and investigated their role in antigen presentation and cellular processes relating to immune responses.

For the first time, we were able to discriminate the roles of the ADF/cofilin family members in primary immune cells. Here we show that n-cofilin is required for antigen presentation through the MHC class II-complex, suggesting that n-cofilin driven actin dynamic plays a critical role for receptor availability and signalling in the immunological synapse. Furthermore, macrophage spreading, control of cell polarity and migration were found to be n-cofilin dependent. Interestingly, ADF is dispensable for all aspects of macrophage motility as well as antigen presentation, however ADF contributes to cell shape control and polarity. These results highlight an unexpected degree of ADF/cofilin specificity in macrophages in controlling cell motility and immune cell function.

## Results

### Cofilin/ADF isoforms in macrophages – n-cofilin is the major macrophage isoform

**Figure 1:**
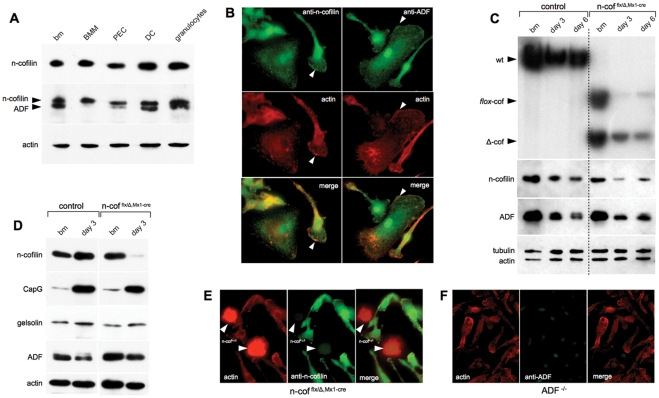
Expression of ADF and n-cofilin in myeloid cells and gene deletion in macrophages. (A) Western blot analysis of different myeloid cell types. In the middle panel, the filter was probed with n-cofilin and ADF specific antibodies to allow a comparison of relative amounts. Comparable levels of n-cofilin were found in total bone marrow (bm), bone marrow derived macrophages (BMM), peritoneal exudate cells (PEC), dendritic cells (DC) and granulocytes. ADF is down-regulated in BMM and PEC. Actin is shown as loading control; (B) Distribution of ADF and n-cofilin in wildtype BMM. ADF and n-cofilin (green, antibodies) co-localize with F-actin rich domains (red, phalloidin for ADF staining, and anti-actin antibody for n-cofilin staining, see arrows); (C) Time course of n-cofilin deletion in bone marrow derived macrophages after poly(I-C) treatment. Deletion on day 0, 3 and 6 of culture. Upper panel: Southern blot analysis for n-cofilin deletion as indicated by the loss of the flx-allele. Wild type (wt), conditional (*flox*-cof) and the deleted alleles (Δcof) are indicated. Lower panels: Western blot analysis of the respective cell lysates after n-cofilin deletion. The amount of n-cofilin strongly decreases on day 3 and slightly increases again at day 6, due to cells, which escaped the deletion. Actin and tubulin are shown as loading controls; (D) Upregulation of the macrophage marker CapG during BMM differentiation, ADF and gelsolin expression is not altered upon deletion of n-cofilin; (E, F) Cell shape of n-cof^null^ and ADF^−/−^ macrophages. (E) N-cof^null^ BMM can be identified in n-cof^flx/Δ,Mx1-cre^ cultures, using an anti-n-cofilin antibody (green). N-cof^null^ BMM had a distinct round shape and showed a high actin signal (red, anti-actin antibody). N-cofilin expressing escaper cells have a regular shape and much lower F-actin content. (F) ADF^−/−^ macrophages (ADF antibody staining, green) have regular morphology, cell polarity and F-actin content (phalloidin, red).

N-cofilin is broadly expressed in most cell lineages, while m-cofilin was found enriched in muscle, and ADF in tissues containing a lining epithelium [23], [25]. A detailed comparison in myeloid cells has not been performed yet. We therefore isolated primary cell lineages of myeloid origin, and determined the levels of ADF and n-cofilin expression in the different cell types. N-cofilin levels were comparable in macrophages, dendritic cells and granulocytes (Fig 1A, upper panel). ADF was mainly expressed in total bone marrow cells (bm), dendritic cells (DC) and in granulocytes (Fig 1A, middle panel). Upon differentiation towards macrophages (BMM, PEC), ADF expression is downregulated, while n-cofilin expression remains constant (Fig. 1A, middle panel).

Quantitative western blot analysis showed that in total bone marrow lysate the ratio of n-cofilin and ADF protein is 1∶1, while in differentiated macrophages (BMM) the ratio changes to about 8∶1 (Fig. S1A). It should be noted that *in vitro* ADF was shown to have a 4-fold higher F-actin depolymerizing activity compared to n-cofilin [25], [26]. Muscle cofilin (m-cofilin) was not detectable in any myeloid cell type using an m-cofilin specific antibody (data not shown). Altogether these data show that within the myeolid lineage, a high degree of specificity in terms of n-cofilin and ADF expression exists.

ADF and n-cofilin were shown to localize to regions of high actin filament dynamics such as lamellipodia [27], and upon cell stimulation they can redistribute to the plasma membrane [28]. Also in primary macrophages we found ADF and n-cofilin mainly at the leading edge, co-localizing with F-actin-rich domains (Fig 1B).

### Deletion of n-cofilin in macrophages results in increased F-actin levels and abnormal cell shape

To examine the role of ADF/n-cofilin in macrophages, we capitalized on mouse mutants carrying a conditional allele for n-cofilin [23], and a conventional knockout strain for ADF [24]. Bone marrow from ADF mutant mice was directly used to culture ADF^null^ macrophages (BMM, bone marrow macrophages). To obtain n-cof^null^ macrophages we first deleted n-cofilin in the bone marrow (*in vivo*) prior to culture. Bone marrow deletion of n-cofilin was achieved using a type I interferon-inducible Mx1-cre mouse, and injection of the type I interferon inducer poly(I-C) (n-cof^flx/Δ,Mx1cre^)[29]. 24 hours after a single poly(I-C) injection, n-cofilin deleted bone marrow cells were taken in culture (see methods).

After 3 days, primary cultures normally contained 70–90% n-cofilin depleted cells as judged by Southern blot analysis and the ratio of the Δcof to flox-cof allele (Fig 1C, upper panel). N-cofilin protein levels were strongly decreased in total cultures (Fig 1C, lower panel and Fig 1D). The residual n-cofilin was due to cells, which had escaped the deletion. Cells with a deleted n-cofilin gene do not express n-cofilin anymore as shown by immunofluorescence (see Fig 1E). The efficiency of differentiation from bone marrow cells towards macrophages was monitored by the increased expression of macrophage markers such as CapG (Fig 1D). The expression of the F-actin severing protein gelsolin did not change during differentiation, while ADF expression decreased (Fig 1D). Mature macrophages were obtained after 6 days in culture. Control and n-cofilin mutant BMM showed a comparable expression of CD11b, MHC class I, MHC class II, and CD86 (Fig S1B). After 6 days in culture the n-cofilin levels in total lysates were slowly increasing again due to the growth advantage of macrophages that had escaped the initial deletion (Fig 1C).

**Figure 2:**
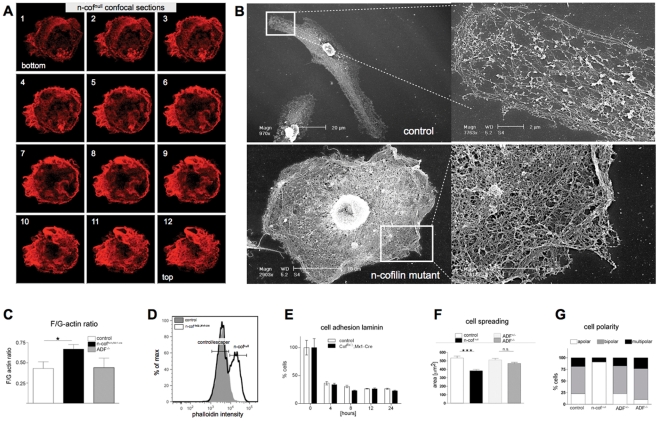
Characterization of n-cofilin null macrophages. (A) Confocal sections through an adherent n-cof^null^ macrophage show the unusual cytoskeletal structure and the impaired leading edge formation. F-actin (in red) is shown by phalloidin staining. Note the short extensions emerging from the cell body and the lack of a lamellipodium; (B) Scanning EM of saponin extracted macrophages illustrate a loose network of short filaments in the lamellipodium of control cells, while n-cof^null^ BMM show densly packed cortical F-actin bundles; (C) Biochemical fractionation showed increased Triton X-100 stable F-actin levels in n-cof^flx/Δ,Mx1-cre^ macrophage cultures (n_culture_ = 6, p<0.05). ADF^−/−^ macrophages showed normal F-actin levels; (D) FACS overlay of phalloidin stained macrophages from a day 6 control (grey histogram) and n-cof^flx/Δ,Mx1-cre^ (black line histogram) culture. About 10-fold increase in phalloidin signal intensity was observed in n-cof^null^ cells, while the escaper cells are represented in the low intensity F-actin peak, similar to the control cells. (E) Cell adhesion on laminin is not altered in n-cofilin mutant macrophage cultures (n-cof^flx/Δ,Mx1-cre^) at 4 to 24 hours after plating. The average of three independent experiments performed in quadruplicates is shown; (F) Cell spreading was significantly impaired in n-cofilin mutant macrophages (n-cof^null^). The spreading area was compared in control (n-cof^wt/wt,Mx1-cre^), ADF^-/-^, ADF^+/−^ and n-cofilin mutant BMM (n_cells_>100, student's test, n-cof^null^:control p<0,0001; ADF^−/−^:ADF^+/−^ p = 0,15); (G) N-cof^null^ BMM fail to polarize. The shape of more than 90% of n-cof^null^ BMM was apolar, while 75% of control and ADF^+/−^ cells were either bipolar or multipolar (n_cells_>130). ADF^−/−^ BMM showed a noticeable tendency to increase cell polarization.

N-cof^null^ macrophages could be distinguished from escaper cells by immunofluorescence using an n-cofilin specific antibody (Fig 1E). One consistent feature of n-cof^null^ macrophages was the round cell shape and the high F-actin content (see also Fig. S1C,D). In contrast, ADF^−/−^ macrophages showed normal F-actin content and a polarized morphology (Fig 1F, 2C). The accumulation of F-actin in n-cofilin mutants was confirmed by biochemical fractionation of G-actin and F-actin in n-cof^flx/Δ,Mx1cre^ cultures (see methods). A 50% increase in the F/G-actin ratio was seen in n-cofilin mutant cultures (Fig 2C). In this assay the F-actin content of n-cof^null^ macrophages is in fact underestimated due to the presence of escaper cells. The high F-actin content of n-cof^null^ macrophages can be better appreciated by FACS analysis, showing a 10-fold increase in phalloidin fluorescence intensity when compared to control cells (n-cof^wt/wt,Mx1cre^) and escaper cells (Fig 2D).

Confocal microscopy of fixed cells revealed that n-cof^null^ macrophages produce short actin-rich protrusions, but that they fail to stabilize a leading edge (Fig 2A). Live microscopy of n-cof^null^ macrophages transfected with Lifeact-GFP confirmed the poor leading edge formation and demonstrated the slow turnover of F-actin structures (see video S1 and Fig S1D).

In fixed macrophages the monomeric actin pool, as highlighted by the staining with DNaseI, did not reveal a major structural difference. DNaseI positive structures were present in control as well as mutant cells and partially localized to F-actin rich domains (Fig S2A).

Electronmicroscopy studies of extracted cells illustrated the pronounced difference in cytoskeletal organization. A very dense accumulation of thick actin bundles was seen in n-cof^null^ macrophages, while control macrophages had a loose peripheral network of crosslinked actin filaments (Fig 2B). The exaggerated stability of actin bundles in n-cof^null^ macrophages, was also illustrated by the resistance of F-actin to saponin extraction (Fig S2B).

The biochemical parameters of n-cofilin and ADF dependent F-actin depolymerization have been investigated in detail [18], [22] and are largely in agreement with our *in vivo* observations. Taken together, the biochemical data, *in vivo* imaging and EM studies presented here indicate that in macrophages actin organization and dynamics largely depend on n-cofilin activity.

Deletion of n-cofilin in macrophages impacts on cell shape parameters, spreading and cell polarity. Adhesion to laminin (Fig 2E), and uncoated plastic (Fig S2C) was normal for n-cof^null^ as well as for ADF^−/−^ bone marrow cells. However, n-cof^null^ macrophages failed to spread efficiently (Fig 2F) and to polarize. This was quantitated, by determining the shape factor. N-cof cof^null^ macrophages had a shape factor of 0.9 (1.0 represents a perfect circle). Interestingly, ADF^−/−^ macrophages showed a significantly lower shape factor (0.4) than control macrophages (0.5), which resulted from a more elongated cell shape (Fig S2D). To further characterize cell polarity, we manually scored cells with a leading edge and uropod as ‘bipolar’, cells with no defined leading edge as ‘apolar’, and cells with multiple lamellipodia as ‘multipolar’. By applying these criteria, more than 90% of n-cof^null^ macrophages were apolar, conversely 80% of control macrophages were either bipolar or multipolar (Fig 2G). In agreement with the shape factor measurements, more ADF^−/−^ macrophages were found polarized when compared to control macrophages (Fig 2G).

The described defects in n-cof^null^ macrophages were scored in resting, non stimulated macrophages. One interesting question was, whether other pathways and actin binding proteins could in principle compensate the lack of n-cofilin. In order to address this question we serum deprived macrophages and then stimulated with M-CSF. Under these conditions control macrophages loose their regular shape and upon M-CSF addition, they start ruffling through a CapG dependent pathway (Fig S2E). N-cof^null^ macrophages on the other hand were resistant to serum starvation and maintained their round shape. Interestingly, M-CSF induced ruffling was impaired, but not completely blunted in mutant cells. This suggests, that M-CSF stimulated actin remodelling can, to a limited extend, bypass the lack of n-cofilin, possibly by the activation of capping protein CapG, which has previously been shown to mediate M-CSF stimulated ruffling [30].

### Macrophage precursor expansion and cytokinesis is n-cofilin dependent

**Figure 3:**
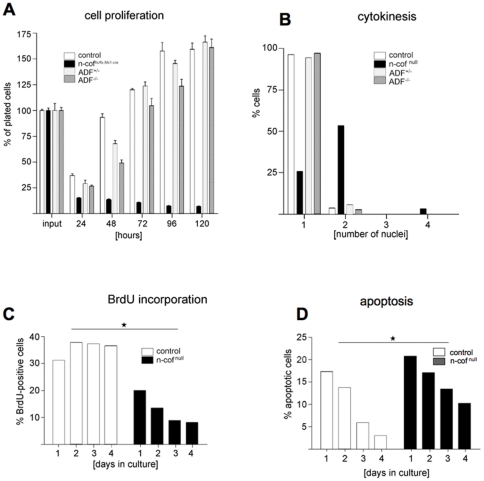
Cell proliferation and cytokinesis is impaired in n-cofilin null macrophages. (A) Cell proliferation was impaired in n-cof^flx/Δ,Mx1-cre^ cultures. Within 120 hours after plating, n-cof^flx/Δ,Mx1-cre^ bm cells did not expand (average of three independent experiments is shown); (B) Cytokinesis defect in n-cof^null^ cells and accumulation of multinucleated cells (n_cells_>150, 2-way ANOVA analysis: Mx1-cre:n-cof^null^ p<0.0001). ADF is not required for cytokinesis; (C) In n-cof^flx/Δ,Mx1-cre^ cultures DNA synthesis decreased during the first days of bone marrow culture (n_cells_>200); (D) TUNEL staining showed slightly increased apoptosis in n-cof^flx/Δ,Mx1-cre^ cultures (n_cells_>100).

Generally bone marrow cells start to expand in culture after a short adaptation phase. However, bone marrow cells from n-cof^flx/Δ,Mx1cre^ mice did not show a significant increase in cell numbers within the first days after seeding (Fig 3A). This could be either due to impaired cell division or increased cell death. FACS analysis with a LIVE/DEAD dye revealed that the general viability of control and mutant cells in the cultures was not significantly altered (Fig S3A).

BrdU incorporation was employed to assess cell proliferation. Control cultures and ADF^−/−^ macrophages showed a relative constant proliferation rate over a 4 day period, while BrdU incorporation gradually decreased in n-cof^flx/Δ,Mx1cre^ cultures (Fig 3C). Due to adaptation and the selective survival of myeloid precursors, apoptosis is usually higher at the beginning of bone marrow culture, and then decreases over time (Fig 3D). In n-cof^flx/Δ,Mx1cre^ cultures apoptosis also decreased over time, however at all time points apoptosis remained slightly elevated.

These data suggest that the observed lack of cell expansion in n-cof^flx/Δ,Mx1cre^ cultures is mainly due to a decreased proliferation rate. This impaired proliferation is most likely due to a cytokinesis defect. It has been previously reported that cofilin accumulates at the contractile ring during cytokinesis [31]. In control cultures (n-cof^wt/wt,Mx1cre^) more than 95% of cells were mononucleated, while only 25% of n-cof^null^ macrophages contained a single nucleus (Fig. 3B). More than 50% of n-cof^null^ macrophages contained two or more nuclei. About 20% of mutant cells were not scored because of an unusually enlarged nucleus with amorphous chromatin structure. Macrophages lacking ADF had no detectable defects with respect to proliferation or cytokinesis (Fig 3A,B).

### N-cofilin is essential for macrophage migration *in vitro* and *in vivo*

**Figure 4:**
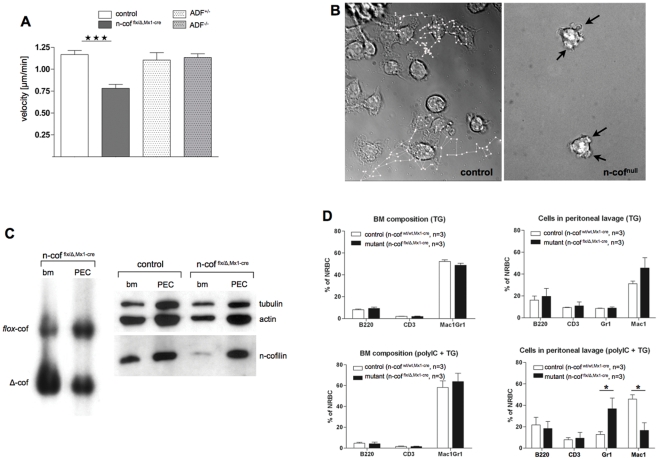
*In vitro* and *in vivo* migration of macrophages lacking n-cofilin. (A) Random motility of n-cof^null^ BMM is impaired, ADF^−/−^ macrophages behave normally. Velocity of center movement over a period of 20 min is shown (n_cells_>60, student's test n-cof^null^:control p<0,0001); (B) Over a period of 8 hours n-cof^null^ cells show no significant net translocation, cell protrusions collapse back onto the cell body (see arrows). Migratory tracks are shown by white lines and dots, for two cells each (see also videos S1, S2, S3); (C) *In vivo* recruitment of n-cof^null^ cells to sites of inflammation is impaired. Thioglycollate injection (TG) into n-cof^flx/Δ,Mx1-cre^ mice does not lead to recruitment of mutant macrophages (Δcof) to the peritoneum (PEC). N-cofilin depleted cells remain in the bone marrow (bm). Southern blot of the respective mutant cell fractions (n-cof^flx/Δ,Mx1-cre^) is shown in the left panel, and the corresponding western blot in the right panel. PEC mainly consists of escaper cells as indicated by the equal Δcof/*flox*-cof signals in Southern blot and the presence of n-cofilin protein. Western blot analysis of control mice (n-cof^wt/wt,Mx1-cre^), is shown on the right panel to illustrate the normal expression levels of n-cofilin in bm and PEC under these conditions; (D) FACS analysis of bone marrow (bm) and PEC from the experiment in (C). Bone marrow composition after TG injection (TG) and n-cofilin deletion (poly(I−C)+TG) was comparable (left panels). The main portion of bm cells were Mac1/Gr1 positive, few B cells (B220) and T cells (CD3) were detected. By FACS the Mac1 and Gr1 positive cells in bm appear as one population. 3 days after TG treatment, the majority of PECs in controls are positive for the macrophage marker Mac1 (upper right panel). Upon deletion of n-cofilin (poly(I−C)+TG), the PEC population increases in Gr1 (granulocytes) and decreases in Mac1 (macrophages).

One important question was, whether impaired leading edge formation and the defects observed in cofilin mutant cultures would also translate to migration defects *in vitro* and *in vivo*. Therefore, macrophages were analysed during 8 hours by time lapse microscopy 24 hours after seeding. The velocity and centre displacement of randomly moving n-cof^null^ macrophages was significantly reduced in comparison to control or ADF^−/−^ macrophages (Fig 4A, B). Stimulation by cytokines had no effect on n-cofilin mutant motility and therefore chemotaxis experiments were not performed. The video sequences (see video S2 and S3) illustrate the random pattern of cell protrusion and retraction and the futile effort to stabilize membrane protrusions (Fig 4B, arrows).

To probe macrophage migration in the tissue context, we established an *in vivo* migration assay that would directly allow us to compare n-cof^null^ cells and control cells (escaper cells) in the same animal, and the same experiment. Untreated mice with one deleted and one floxed allele (cof^flx/Δ,Mx1cre^) yield a 1∶1 ‘Δcof/flx-cof’ signal ratio in Southern blot. We then deleted n-cofilin in the bone marrow by applying a single dose of poly(I-C). Deletion – equivalent to the generation of the Δcof allele – was scored by the Southern blot signals. 48 hours later, thioglycollate was injected into the peritoneum to induce a mild inflammatory reaction and the recruitment of immune cells (PEC). Infiltration generally occurs in two waves, with granulocytes arriving first [32], followed by macrophages, accounting for more than 90% of PEC after 5 days [33]. This assay allowed us to compare the recruitment of n-cof^null^ cells (Δcof/Δcof) and control cells (flox-cof/Δcof) from the bone marrow to the peritoneum. 3 days after thioglycollate injection about 60% of bone marrow cells were n-cof^null^ cells, while in the peritoneum only 5% of cells were n-cofilin mutants (Δcof/Δcof). 95% of PEC were n-cofilin expressing flox-cof/Δcof cells (Fig 4C, left panel). Western blot analysis confirmed that in mutants, cofilin protein depletion was indeed restricted to the bone marrow (Fig. 4C right panel). This result shows that *in vivo*, n-cofilin is essential for macrophage recruitment to inflammatory sites. To account for potential secondary effects due to interferon induction, all control animals (cof^wt/wt,Mx1cre^) received poly(I-C) injections as well.

In control mice poly(I-C) injection did not significantly affect the composition of the bone marrow or PEC as determined by B220, CD3, Gr-1 and Mac-1 expression (Fig 4D). However, when n-cofilin was deleted, the PEC population showed a significant bias towards granulocytes (Fig 4D, lower right panel). This finding is consistent with an early granulocytes infiltration before n-cofilin deletion is complete, while the second wave of Mac1-positive n-cof^null^ macrophages does not reach the peritoneal cavity.

### Role of n-cofilin in immune responses

**Figure 5:**
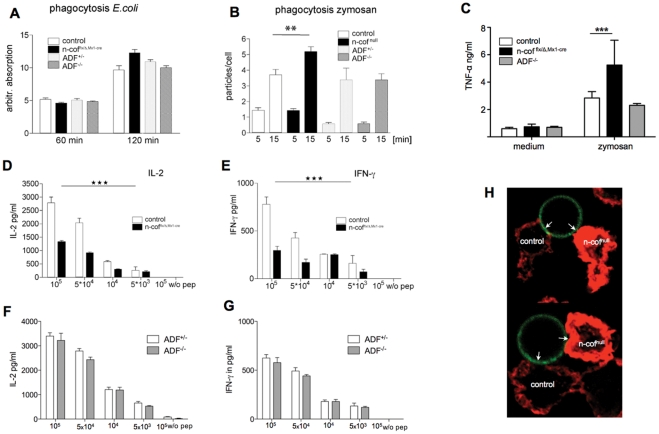
Immunological responses of n-cofilin mutant dendritic cells. (A) Phagocytosis of FITC-labelled *E. coli* in n-cof^flx/Δ,Mx1-cre^, ADF^-/-^, ADF^+/-^, and control macrophage cultures (n-cof^wt/wt,Mx1-cre^); (B) Phagocytosis of FITC-zymosan by individual macrophages was scored (n_cells_>100). Phagocytosis in ADF^−/−^ cells was not changed; (C) TNF-α production induced by Zymosan phagocytosis. Macrophages were allowed to ingest zymosan particles and after 16 hours, TNF-α levels were determined in the cell supernatant; (D-E) Impaired T-cell stimulation by n-cofilin mutant dendritic cells. Serial dilutions of OVA_323-339_ peptide were presented by n-cof^fwt/wt,Mx1-cre^ or n-cof^flx/Δ,Mx1-cre^ dendritic cells to OT-II T-cells. T cell stimulation was measured by production of IL-2 (D) and IFN-γ (E) after 24 hours; (F-G) T-cell stimulation of ADF^−/−^ dendritic cells was similar to ADF^+/−^ controls; (H) MHC class II-induced contact formation is impaired in n-cof^null^ macrophages. A mix of control macrophages (escaper cells) and n-cof^null^ macrophages, preincubated with anti-MHC class II antibody were allowed to form contacts with the beads. Control cells form F-actin free contact zones (arrows) with anti-mouse-IgG-coated beads (FITC labeled, green), while n-cof^null^ macrophages showed a F-actin rich domain (phalloidin, red) at the interface with the bead (arrows).

Phagocytosis, antigen processing, and subsequent antigen presentation to T-cells are important immunological functions of BMM and DC [34]. To analyse the role of n-cofilin in phagocytosis, we followed the uptake of *E. coli* particles and of zymosan. Interestingly, phagocytosis was not impaired in n-cof^flx/Δ,Mx1cre^ and ADF^−/−^ macrophages. On the contrary, bacteria uptake was slightly increased in n-cofilin mutant cultures at 120 min (Fig 5A, p = 0.84). The uptake of FITC-zymosan particles was scored manually for individual macrophages. While after 5 minutes, no difference in the number of internalized particles was seen among the different genotypes (Fig 5B), after 15 minutes n-cof^null^ macrophages had taken up significantly more yeast particles when compared to control and ADF^−/−^ macrophages. In agreement with this finding, formation of an F-actin-rich phagocytic cup around the engulfed particles was not dependent on n-cofilin (Fig. S3B).

To reassure that particles were indeed internalized and that phagosomes fused with the lysosomal compartment, we performed another uptake experiment using pHRodo-zymosan. Upon fusion of pHRodo-zymosan containing endosomes with the acidic lysosomal compartment, pHRodo-zymosan turns fluorescent. Also in this assay, uptake was more efficient in cof^null^ macrophages (Fig. S3C). Taken together these findings suggest, that despite the reduced actin dynamics, n-cof^null^ cells can efficiently engulf particles by phagocytosis, and trigger the production of TNF-α (Fig 5C). Interestingly, n-cofilin mutant macrophages also produced increased amounts of TNF-α when compared to control or ADF^−/−^ cells.

The subsequent step of proteasomal antigen-processing was tested using DQ-ovalbumin, which yields fluorescent peptides upon proteolytic degradation. In n-cofilin mutant macrophage cultures DQ-ovalbumin was processed normally as shown by FACS analysis of the produced fluorescent peptides (Fig S3D), suggesting that pinocytosis and antigen processing are not n-cofilin dependent.

To finally induce T cell activation, peptides must be presented to CD4^+^ T-cells via the MHC class II complex. T-cells have to make physical contact with MHC class II harbouring antigen presenting cells through the immunological synapse [35]. The structure and dynamic changes within this contact zone and lateral mobility of signalling molecules are thought to play an important role in T-cell activation [36].

The ability of n-cof^null^ cells to present antigen was tested using MHC class II mediated presentation of the ovalbumin peptide OVA_323-339_ and stimulation of OT-II T-cells. T cell activation was then assessed by their release of IL-2 and IFN-γ from the T-cells [37]. In this experiment, cells from n-cof^flx/Δ,Mx1cre^ mice were severely impaired in stimulating IL-2 and IFN-γ production (Fig 5D, E). MHC-II expression of MHC-II and CD86 on the surface was found to be comparable in n-cof^null^, and control cells (see Fig S1B), excluding the possibility that lack of surface receptor representation could account for the impairment in T-cell activation. ADF^−/−^ cells on the other hand were indistinguishable from controls in activating T-cells (Fig 5F, G).

Cell-cell contact formation controls the efficacy of T cell priming and we thus hypothesized that contact formation between n-cof^null^ antigen presenting cells and T cells might not be productive for T cell activation. To investigate this hypothesis, we employed a model to study MHC class II mediated contact formation of antigen presenting cells with beads [38].

When we tested control and n-cof^null^ cells to make contact to the beads, both were able to bind to the beads (Fig 5H), however the architecture of the contact zones and the F-actin distribution was very different between control and n-cof^null^ cells. While control cells were able to form a tight contact zone along the perimeter of the beads that was virtually free of filamentous actin (Fig 5H, see arrows), n-cof^null^ cells formed an irregular contact zone with gaps and a strong accumulation of F-actin along the contact site with the bead (see arrows). The different topology of contact zones suggests, that n-cof^null^ antigen presenting cells fail to form productive cell-cell contacts, which could explain the poor activation of T-cells as observed in Fig 5 D,E.

## Discussion

The biochemical aspects by which the ADF/cofilin protein family can regulate actin filament dynamics and chemotaxis have been studied in detail [18], [22], [39], however the physiological role of ADF/cofilin is just beginning to unravel [23], [24]. One major unresolved question concerns the specificity of the three ADF/cofilin proteins. ADF, n-cofilin and m-cofilin are interchangeable in biochemical assays, in terms of F-actin severing, and F-actin depolymerization activity [40], [41]. The regulation of the three ADF/cofilin proteins appears to use common mechanisms such as phosphorylation of Ser3 [21], [42]; [43]. However, our genetic studies revealed that fundamental differences must exist among the isoforms in linking actin dynamics to cell function – in particular in macrophages and dendritic cells.

Our major aim was to focus on the functions and differences of ADF/cofilin in antigen presenting cells. In general, the role of ADF/cofilin in authentic immune cells is poorly understood. Detailed studies on immortalized cell lines (e.g. HL-60, U937, J774.1, Jurkat, HL-60,) have suggested a role of ADF/cofilin family members in chemotaxis, phagocytosis, respiratory burst, but also T-cell activation via the TCR [28], [44], [45]. However, the specificity of ADF and n-cofilin in these processes has remained unclear as well as the relevance in authentic primary immune cells.

We employed a genetic approach to address this important question in primary mouse macrophages and concentrated on cell functions related to the immune response. In order to study n-cofilin function in bone marrow derived macrophages, we had to design a novel strategy for gene deletion by using an Mx1-Cre driver line. Bone marrow specific deletion by multiple injections of poly-(IC) could not be employed, because the known deletion pattern of Mx1-cre in other tissues would cause lethality upon deletion of n-cofilin. Therefore we developed a protocol where a single injection led to efficient bone marrow deletion of n-cofilin, just after 24 hours. This novel strategy should be very useful for analysing other essential genes, which lead to a similar severe phenotype as n-cofilin deletion.

A number of unexpected findings emerged from our studies on bone marrow derived macrophages, highlighting novel functions of the ADF/cofilin family of proteins.

ADF deletion in macrophages did not impair any of the tested physiological responses. For example, F-actin levels were not altered and migratory behaviour of macrophages was normal. However, ADF^null^ macrophages showed an exaggeratedly elongated cell shape when compared to control macrophages. On the contrary, deletion of n-cofilin basically abolished cell polarization. One possible explanation for these opposing findings is that ADF and n-cofilin may use distinct signalling pathways to control actin dynamics, the second explanation could simply be a dose effect, since in macrophages ADF expression is lower than n-cofilin. The published biochemical data and our expression analysis would rather argue for the first explanation. ADF has been shown to have a 4-times higher F-actin severing activity when compared to n-cofilin [25], [26], and dendritic cells express similar levels of ADF and n-cofilin (see Fig 1A and Fig S1A). Nevertheless, n-cof^null^ dendritic cells accumulate F-actin, while ADF^null^ cells show normal F-actin levels and normal antigen presentation.

Also, if we further reduce the overall F-actin depolymerizing activity in ADF^null^ macrophages by deleting one of the n-cofilin alleles, these compound mutant macrophages (ADF^-/-^/n-cof^flx/wt,Mx1-cre^) have normal cell shape and normal F-actin content (Fig S3E).

Taken together these findings argue against a simple 'dose effect' of ADF and n-cofilin, and rather support the notion that both molecules must have, not yet identified distinct or additional activities, which are not revealed in actin based biochemical assays [23], [24]. The different phenotypes of ADF and n-cofilin mouse mutants during embryonic development also support the notion that both proteins work in distinct pathways [23], [24].

One prediction of this hypothesis would be, that the phenotype of ADF^null^ and n-cof^null^ macrophages should be qualitatively different, rather than show incremental differences. In fact, the latter is the case, e.g n-cof^null^ macrophages are lacking cell polarity, while ADF^null^ macrophages show the opposite phenotype – a more pronounced polarity and extended cell shape. The mechanistic basis of the defects observed in n-cof^null^ macrophages is the strong accumulation of filamentous actin and the lack of dynamic turnover. We documented this defect by F-actin measurements, electronmicroscopy and live imaging. More quantitative methods to measure actin dynamics such a FRAP were not possible in n-cof^null^ macrophages, due to the round and heaped up cell structure.

How can we reconcile these data with our current knowledge of ADF, n-cofilin activity? One speculation is that ADF might be involved in the regulation of actin-myosin interaction and ultimately cell contraction, due to the described antagonizing activities of ADF/cofilin and tropomyosin [46]. Deletion of ADF could then mainly affect the alignment of actin filaments and cell contractility, rather than controlling actin filament length and F-actin levels. In this model, n-cofilin would then account for actin filament severing and filament turnover.

Along the same lines it might be interesting to re-visit other mouse models for F-actin regulators. Apart from ADF and n-cofilin, macrophages also express the F-actin severing protein gelsolin, and its close homolog CapG [30]. Interestingly, both proteins are not sufficient to compensate for the loss of n-cofilin, although *in vitro* gelsolin is the predominant actin severing protein [47].

Conversely, we were surprised that phagocytosis was not impaired in n-cof^null^ macrophages despite their increased F-actin levels and the cell polarity defects. This is in agreement with previous reports showing that phospho-n-cofilin has a stimulatory role in phagoytosis and that n-cofilin knockdown using anti-sense oligonucleotides enhances phagocytic activity [28], [48]. Apparently, other parameters of actin turnover such as F-actin capping and uncapping are more important for the efficacy of phagocytosis. Indeed results from gelsolin^null^ and CapG^null^ macrophages have shown that filament capping by CapG, but not F-actin severing by gelsolin is crucial for phagocytosis [30], [49]. This might also explain our observation in n-cof^null^ macrophages.

One common theme that emerges from the here presented results, published data on neurons [24], and observations on stem cells (Gurniak & Witke, unpublished) is a critical role of ADF/cofilin in controlling cell polarity. Impaired cell polarity will translate into cell division defects as well as migration defects. Efficient cell polarization might also be required for the final steps of antigen presentation. Macrophages and dendritic cells support the spatial re-orientation of MHCII molecules organized in lipid rafts towards the immunological synapse, which allows sustained MHC class II/TCR interactions for subsequent T-cell activation [50], [51], [52]. A recent report showed for the first time the necessity of cytoskeletal remodelling on the APC side for efficient T cell priming [53]. It is thus conceivable that the rigid actin cortex in n-cof^null^ cells could interfere with the movement of microdomains and lipid rafts in the membrane and thus render it more difficult for the T cell to form stable contacts with sufficient peptid-loaded MHCII molecules. In fact, reduced receptor mobility has been reported at synapses of neurons devoid of n-cofilin [54].

The data suggest that n-cofilin acts as the key factor to resolve the cortical actin network during contact formation of dendritic cells with T-cells. This requires that adhesion molecules diminish the distance between the two cells to approximately 13 nm and an extensive interdigitation of the glycocalix [55]. One can speculate that this process requires a certain membrane flexibility on both sides and that the lack of n-cofilin leads to inefficient alignment of the cells. The static cortical F-actin network, which we observed in n-cof^null^ cells locks the cells in a conformation, and prevents the formation of a tight contact zone and activation of T-cells. The reduced surface spreading of n-cof^null^ cells is in agreement with impaired contact formation.

The work presented here stimulates a number of important questions for future work on n-cofilin, and actin in general, in antigen presentation. What is the structure and dynamics of the immunological synapse with respect to actin filament turnover and n-cofilin activity? What are the signalling pathways that control n-cofilin in dendritic cells? What are the downstream signalling pathways in T-cell activation that depend on n-cofilin?

To date most studies have concentrated on the role of ADF/cofilin and actin in T cells [8], [56], however our data, and other studies [38], [50], suggest that actin remodelling in APCs is equally important in forwarding the stimulation signal. With the genetic tools in hand, we can now address the details from the APCs as well as the T-cell side. Here, we identified actin remodelling by n-cofilin as a novel checkpoint for antigen presentation, immunological synapse formation, and T-cell activation.

## Methods

### Mice

ADF knockout mice (ADF^-/-^) and conditional n-cofilin mutants have been described elsewhere [24]. The conditional n-cof^flx^ allele was crossed into the type I interferon-inducible Mx1-Cre line on a C57BL/6 background [29]. Mutant mice with one deleted and one conditional allele were used (n-cof^flx/Δ,Mx1-cre^) to increase deletion efficiency. Control animals carried the wild type allele and the Mx1-cre transgene. Treatment of mice was in accordance with the laws for conducting animal experiments and followed the NIH guide for the care and use of laboratory animals. Permission for experiments was granted by the Italian health ministry, through 'Decreto n. 19/2005-B'.

### Bone marrow culture and macrophage and dendritic cell differentiation

For culture of BMM, cells were seeded at 2x10^5^ cells/cm^2^ in 10 cm^2^ petri dishes or on glass cover slips in BMM medium, consisting of DMEM (Gibco), 1 mM sodium pyruvate (Gibco), 1% Hepes, 1% nonessential amino acids (Gibco), and 10% heat inactivated foetal bovine serum (PAA) supplemented with 25% L929-cell-conditioned medium as a source for M-CSF. Unless specified, all experiments were performed on day 6 of culture. To obtain bone marrow-derived dendritic cells (DC) this protocol was slightly modified. Bone marrow cells were cultured in RPMI1640 with 10% FCS supplemented with 20 ng/ml GM-CSF and harvested after 7 days of culture. To induce cre-recombinase expression in mutant n-cof^flx/Δ,Mx1-cre^ and control n-cof^wt/wt,Mx1-cre^ animals, a single i.p. injection of 300 μg polyinosinic-polycytidylic acid (poly(I-C) was applied. 24 h later, bone marrow was flushed from the femur and tibia, and cells cultured as described above.

### Cell transfection and analysis of live actin dynamics

On day 5 of culture BMM macrophages were harvested, washed and transfected using a Amaxa® Mouse Dendritic Cell Nucleofector® Kit (Lonza). In brief, 2 μg of LifeAct-GFP plasmid DNA was mixed with 10^6^ BMM resuspended in 100 μl Nucleofector® Solution, before electroporation was done using an AMAXA transfector (programm Y-001). Cells were resuspended in BMM medium and plated on tissue culture treated ibidi plastic slides 24 h–48 h before analysis (Integrated BioDiagnostics). Video sequences of living cells were acquired at 30 frames/min for 5 minutes at 37°C using a Widefield Axiovision inverted microscope (Zeiss).

### Western blot analysis

Total cell extracts were subjected to 15% SDS–PAGE, transferred to Immobilon-P membrane, and probed with the respective antibodies. Anti-n-cofilin (KG40, rabbit polyclonal antibody, were raised against recombinant mouse n-cofilin), anti-ADF (GV-13, Sigma), anti-actin (C-4, MP Biomedicals), anti-gelsolin (ab2480, raised against recombinant mouse gelsolin), anti-CapG (abWW12, raised against recombinant mouse CapG), α-tubulin (DM1A, Sigma). Signals were detected using HRP-coupled secondary antibodies (Molecular Probes) and Amersham-ECL reagent.

### Quantification of G- and F-actin

The Triton-X100 insoluble F-actin fraction was separated from the soluble G-actin content, and the amount quantified. Cells were lysed in ice-cold PHEM buffer (60 mM Pipes, 20 mM HEPES, 10 mM EGTA, 2 m MgCl_2,_ pH 7.0, 1% Triton-X100). After 15 min on ice, F-actin was pelleted by centrifugation for 10 min (Eppendorf swing-out rotor, 4°C, 10.000 rpm). Supernatant, containing G-actin, was collected, the F-actin pellet washed twice with cold PHEM buffer and then dissolved in 1x SDS sample buffer. Equivalent amounts were separated by SDS-PAGE, and F- and G-actin determined by Western blot and densitometry.

To quantify F-actin by FACS analysis BMM were harvested on day 6, fixed in 2% freshly prepared paraformaldehyde, permeabilized and stained with Phalloidin Alexa680. Data acquisition was performed using a FACS Canto (Becton Dickinson), data were by analysed using FlowJo software (Treestar).

### Immunofluorescence

Cells were fixed in 4% freshly prepared paraformaldehyde and stained following standard protocols. Phalloidin Alexa594 (Molecular Probes) was used to visualize F-actin and Hoechst33342 for nuclear staining. For n-cofilin staining using the KG60 antibody, a slightly modified fixation protocol was applied. Cells were fixed in 15% picric acid/4% paraformaldehyde. Under these conditions phalloidin staining could not be applied, and instead the anti-actin antibody C-4 was used to visualize cellular actin. In our hands the C-4 actin staining largely overlapped with a regular phalloidin staining, and therefore nicely highlighted the F-actin structures in cells. As secondary antibodies the respective Alexa488 and Alexa594-labelled antibodies were used (Molecular Probes).

For surface marker expression, differentiated macrophages were harvested, fixed and incubated with the respective surface markers (all BD Biosciences). For quantitation of life versus dead cells the LIVE/DEAD kit was used (Molecular Probes). Data acquisition was performed using a FACS Calibur and a FACS Canto (Becton Dickinson), data were by analysed using FlowJo software (Treestar).

### Scanning EM

For scanning EM of the cytoskeleton cells grown on glass coverslips were incubated in PEG-GTX (10 mM PIPES, 50 mM EDTA, 10 mM KOH, 27 mM KCl, 0.1% (vol/vol) Triton X-100, 4% (vol/vol) polyethylenglycol (MW 6000), 10% (vol/vol) glycerin, pH 7.2) for 5 min to remove the plasma membrane, washed briefly in PBS, and fixed with 4% (vol/vol) glutaraldehyde in PBS. Subsequently cells were dehydrated through a graded series of ethanol and critical point dried from CO_2_ in 10 cycles using a Balzers CPD 030 (BAL-TEC, Schalksmühlen, Germany). Dried specimens were mounted on aluminum sample holders and coated with a 2-nm layer of platinum/palladium in a HR 208 sputter coating device (Cressington, Watford, UK). SEM was performed at an acceleration voltage of 3 kV using an XL 30 SFEG (Philips, Eindhoven, Netherlands) equipped with a through lens secondary electron detector.

### Cell attachment, proliferation assay, apoptosis

In order to assess attachment of n-cofilin depleted bone marrow cells, mice received a single injection of 300 μg poly(I-C) three days prior to harvest. 1x10^5^ bone marrow cells per well were plated in 48-well plates, all measurements on different substrates were performed in quadruplicates. For normalization the total number of seeded cells was determined on the plate. Cell number was measured using the CyQuant Proliferation Assay (Molecular Probes). Samples were analyzed at 485(Abs)/538(Em) nm using a Fluoroskan plate reader. BrdU incorporation was measured using the 5'-Bromo-2'-deoxy-uridine labelling and detection kit II (Roche). At least 200 cells per genotype and time point were counted. Apoptotic cells were detected using the DeadEnd Fluorometric TUNEL System (Promega).

### Migration Analysis

Cells were seeded on tissue culture treated ibidi plastic slides 24 h before analysis (Integrated BioDiagnostics). Video sequences of living cells were acquired at 4 frames/min in controlled atmosphere (37°C/5% CO_2_) using an inverted microscope (Axiovert 100, Zeiss). MetaMorph (Molecular Devices) software was used for acquisition and analysis of the data. For *in vivo* recruitment from the bone marrow to the peritoneum mice received a single injection of 200 μg poly(I-C) i.p. at day -5 to induce n-cofilin deletion in the bone marrow. At day -3 macrophage recruitment was induced by a single i.p. injection of 500 μl 3% thioglychollate and at day 0 PEC and bone marrow were harvested for Southern, Western blot or FACS analysis.

### Phagocytosis, antigen processing and surface marker analysis

1x10^5^ cells in 48-well plates were incubated with 100 μl of FITC-labelled *E.coli* particles and phagocytosis measured according to the manufacturer's instruction (Vybrant Phagocytosis Assay, Molecular Probes). Quadruplicate samples were analysed using a Fluoroskan plate reader.

For uptake of yeast particles, fluorescently labelled FITC-zymosan was incubated with adherent macrophages for various times. Uptake was stopped by washes with ice-cold PBS and fixation in 4% PFA/PBS. Cells were counterstained with Phalloidin-Alexa594 and the number of fluorescent particles per cell was then scored for 100 cells per genotype and time point. Phagocytosis was expressed as the mean of particles per cell. Pictures of phagocytic cups were taken after fixation and phalloidin staining, using a Leica SP5 confocal microscope.

To measure TNF-α production triggered by phagocytosis, FITC-zymosan was added to 5x10^4^ BMM (in 100 μl BMM medium) and culture supernatants were collected after 16 hours. Cytokine levels were determined using a mouse inflammatory cytokine 4-plex panel (Invitrogen) acquired on a Luminex100 (Applied Cytometry) and analysed StarStation software.

Antigen processing and surface marker expression was analysed by FACS. Cells were incubated with freshly prepared DQ-ovalbumin (Molecular Probes) for 0, 10, 60 or 240 minutes to allow uptake and proteolytic processing. Cells were then washed, fixed in 4% PFA/PBS, and analyzed using a FACS Diva.

### T-cell activation assay

T-cells from OT-II mice expressing a transgenic T-cell receptor specific for OVA_323–339_/I-A^b^ [37] were purified from spleen using the PAN T-cell kit (Miltenyi). For T-cell stimulation, 1x10^5^ T cells were co-cultured with different numbers (5x10^3^ – 1x10^5^) of DCs in the presence of 0.3 µg/ml OVA_323–339_ peptide. After 24 hours, the amounts of IL-2 and IFN-γ were determined in the supernatants using DuoSet ELISA development kits (R&D Systems).

### Bead contact assay

Carboxylated beads (10 μm, Polysciences) were coated o.n. at 4^o^C with Alexa488-labeled anti-mouse IgG (Molecular Probes). BMM were incubated for 45 min on ice with an anti-MHC class II antibody (BD Bisciences). Beads and macrophages were mixed (ratio 2∶1) and allowed to form clusters in suspension for 15 min on ice. The suspension was plated on poly-L-lysine coated cover slips, where cells were allowed to attach for 10 min at 4°C. The slides were incubated for another 5 minutes at 37°C, before cells were fixed and imaged using a Leica SP5 confocal microscope.

### Morphometric cell analysis

The shape factor (sf) was calculated using the MetaMorph module “regional measurements” (sf  =  4πA/P^2^, with A: area and P: perimeter).

### Statistical analysis

We used student's t-test for comparison of repeated measurements between two genotypes and 2-way ANOVA with Bonferroni post-test to analyse variances between two genotypes over time. A p-value of <0,05 is indicated by *, a p-value of <0,005 by ** and a value of <0,0005 by *** respectively.

## Supporting Information

Figure S1 (A) Expression of n-cofilin and ADF in bone marrow and bone marrow derived cells. Recombinant GST-fusion proteins were titrated against lysate from total bone marrow (bm) and bone marrow derived macrophages after 6 days of culture (BMM). Quantitative analysis (histogram, lower panel) shows a 1∶1 ratio of n-cofilin and ADF in bm, while in BMM the ratio changes to about 8∶1; (B) Expression of macrophage surface markers on BMM after 6 days of culture. Macrophages from n-cof^flx/Δ,Mx1-cre^ and control mice expressed comparable levels of CD86, MHC class II, and CD11b, the escaper cell population is characterized by a normal phalloidin signal; (C) Morphology of n-cof^null^ macrophages by scanning electron microscopy. Mutant cells showed ruffles but no leading edge, and poor cell polarity (lower panel). Control macrophages had the typical flat morphology, membrane ruffles and extended lamellipodia (upper panel). Images were taken at 5000x magnification; (D) Cytoskeletal dynamics of n-cof^null^ macrophages. Control (C) and n-cof^null^ macrophages (M) were transfected with LifeAct-GFP to visualize actin dynamics in live cells. Video sequences illustrate the high dynamics of actin rich extensions in control cells, while n-cofilin mutant macrophages show very limited actin remodelling (see video S1, S2, S3).
(PDF)

Figure S2 (A) Phalloidin and DNaseI staining of fixed control and n-cofilin mutant macrophages. Note that the exposure times for the phalloidin stained n-cofilin mutant cells (n-cof^null^) had to be reduced to 1/5 of the controls in order to avoid overexposure. The same magnification of control and n-cof^null^ cells is shown; (B) ‘Actin Footprint’ structure of control and n-cof^null^ macrophages. Live macrophages were extracted with 0.05% saponin, after fixation in 4% PFA actin structures were stained with phalloidin (green). In control macrophages only the actin-rich contact sites were preserved, while in n-cof^null^ macrophages most of the cytoskeleton was resistant to extraction; (C) Adhesion of bone marrow derived cells on plastic. Bone marrow cells from mutant macrophages (n-cofilin^flx/Δ,Mx1-cre^), as well as ADF^−/−^ macrophages showed normal attachment to plastic surface; (D) Cell shape as expressed by the ‘shape factor’ (SF). SF = 4ΠA/P^2^ (A: area, P: cell perimeter). A SF of 1 describes a perfect circle, lower values are a measure of cell extension. N-cof^null^ macrophages were almost round (SF = 0,89±0.01), while ADF^−/−^ macrophages were significantly more elongated (SF = 0.38±0.01) when compared to control cells (SF = 0.49±0.02) and ADF^+/−^ cells (SF = 0.53±0.02). n_cells_>100, student's test: n-cof^null^:control p<0,0001; ADF^−/−^:ADF^+/−^ p<0,0001; (E) Starved control and n-cof^flx/Δ,Mx1-cre^ BMM were stimulated for 5 min with 0.5 µg/ml M-CSF. Cells were labelled with phalloidin (green)/DAPI (blue). Note that n-cof^null^ macrophages (white arrows) are resistant to serum deprivation, while control macrophages start retracting cell protrusions. Upon M-CSF stimulation, control BMM re-form cell extensions. Mutant BMM also responded with increased cell spreading.
(PDF)

Figure S3 (A) Viability of control and n-cof^flx/Δ,Mx1-cre^ macrophages, differentiated for 6 days in culture, was shown by staining with violet LIVE/DEAD (see methods). Respective percentages of cells represented in the quadrants are indicated. In the n-cof^null^ and escaper population more than 95% of cells were viable; (B) Phagocytosis of pHRodo-zymosan partricles. pHRodo-zymosan was offered for 15 and 30 min to control and n-cof^flx/Δ,Mx1-cre^ macrophages. Uptake was stopped by washing cells with cold PBS. FACS analysis was used to quantify the uptake of particles into the lysosomal compartment. Two independent experiments each are shown for the control (n-cof^wt/wt,Mx1-cre^) and mutant macrophage cultures (n-cof^flx/Δ,Mx1-cre^). (C) Phagocytic cup formation occurs in n-cof^null^ macrophages (lower panel). After 15 minutes of FITC-labelled zymosan uptake (green), cells were fixed and counterstained for F-actin (phalloidin, red) to visualize the formation of the F-actin-rich cup (see arrows). Nuclei are shown in blue (DAPI); (D) N-cofilin^flx/Δ,Mx1-cre^ macrophages are able to process antigens such as DQ-ovalbumin. Control and n-cofilin^flx/Δ,Mx1-cre^ macrophages were allowed to internalize and process DQ-ovalbumin for 10, 60 and 240 minutes followed by FACS measurement of the fluorescence increase due to the generation of peptides from proteolyzed DQ-ovalbumin. Note that the kinetics of processing are slightly faster in n-cof^null^ macrophages; (E) Specificity of n-cofilin and ADF activity in macrophages. Complete deletion of n-cofilin rather than a general decrease of ADF/cofilin activity is responsible for the observed phenotype. The cell shape of compound mutants (ADF^-/-^/n-cofilin^flx/wt,Mx1-cre^) lacking both alleles of ADF and one allele of n-cofilin is comparable to control macrophages.
(PDF)

Video S1 **Actin dynamics in LifeAct-GFP transfected mutant and control macrophages.** Video sequences of living cells were acquired for 5 minutes at 30 frames/min in a temperature-controlled environment (37°C) using an inverted microscope (Axiovert 200M, Zeiss) with Axiovision software. ImageJ was used for processing of the data.
(MOV)

Video S2 **Shows random migration of control macrophages.** Video sequences of living cells were acquired for 8 hours at 4 frames/min in controlled atmosphere (37°C/5% CO_2_) using an inverted microscope (Axiovert 100, Zeiss). MetaMorph (Molecular Devices) software was used for acquisition and processing of the data.
(MOV)

Video S3 **Shows random migration of n-cofilin mutant macrophages.** Video sequences of living cells were acquired for 8 hours at 4 frames/min in controlled atmosphere (37°C/5% CO_2_) using an inverted microscope (Axiovert 100, Zeiss). MetaMorph (Molecular Devices) software was used for acquisition and processing of the data.
(MOV)
