# Supplementary figures and images for: Immunological Responses and Actin Dynamics in Macrophages Are Controlled by N-Cofilin but Are Independent from ADF
Source: PLoS One. 2012 Apr 27;7(4):e36034. doi: 10.1371/journal.pone.0036034 (PMC3338623; doi:10.1371/journal.pone.0036034)

Supplementary Figure 1: Jönsson et al.

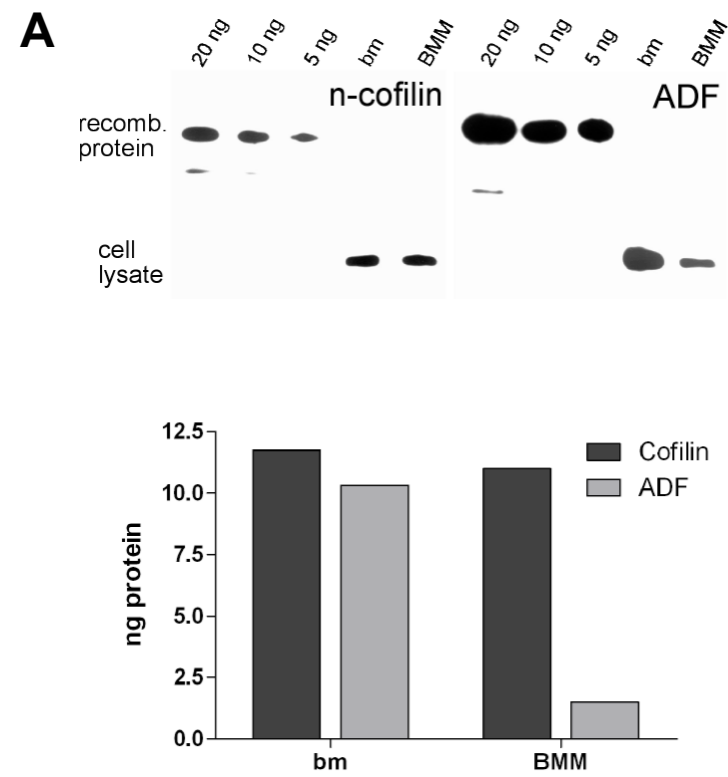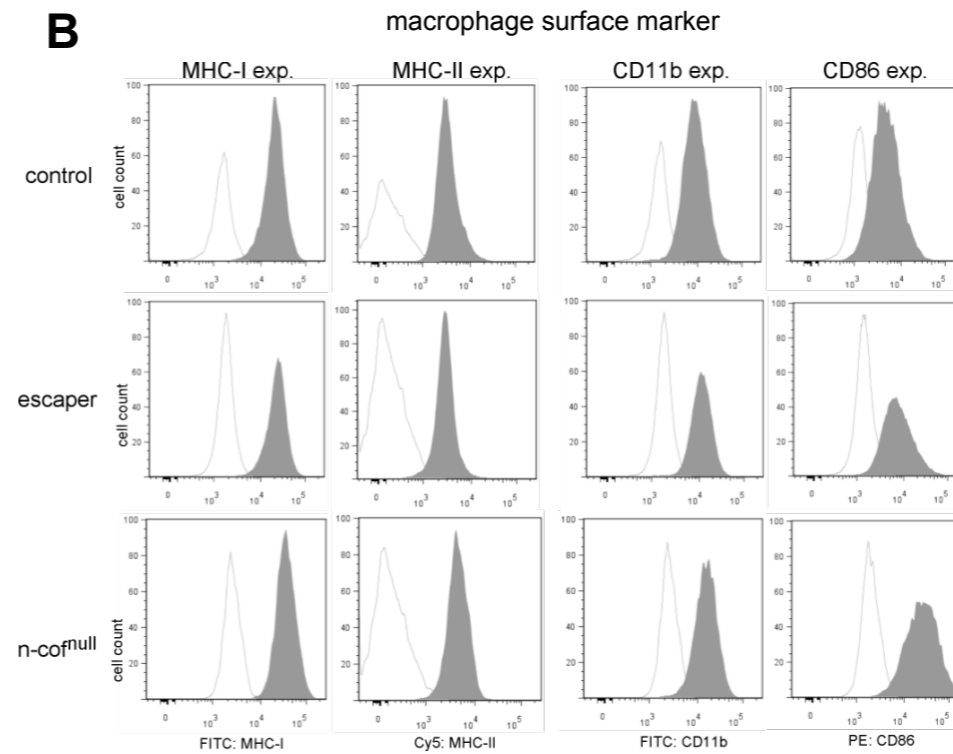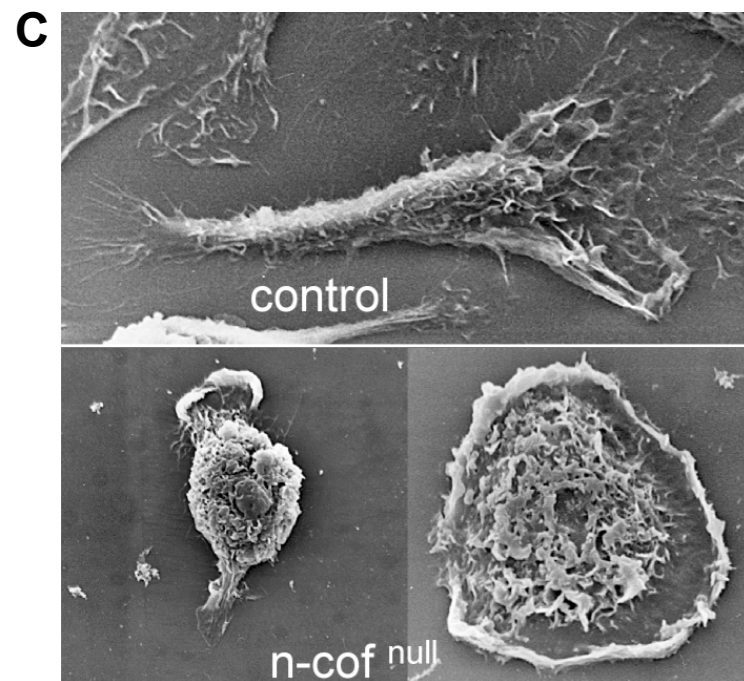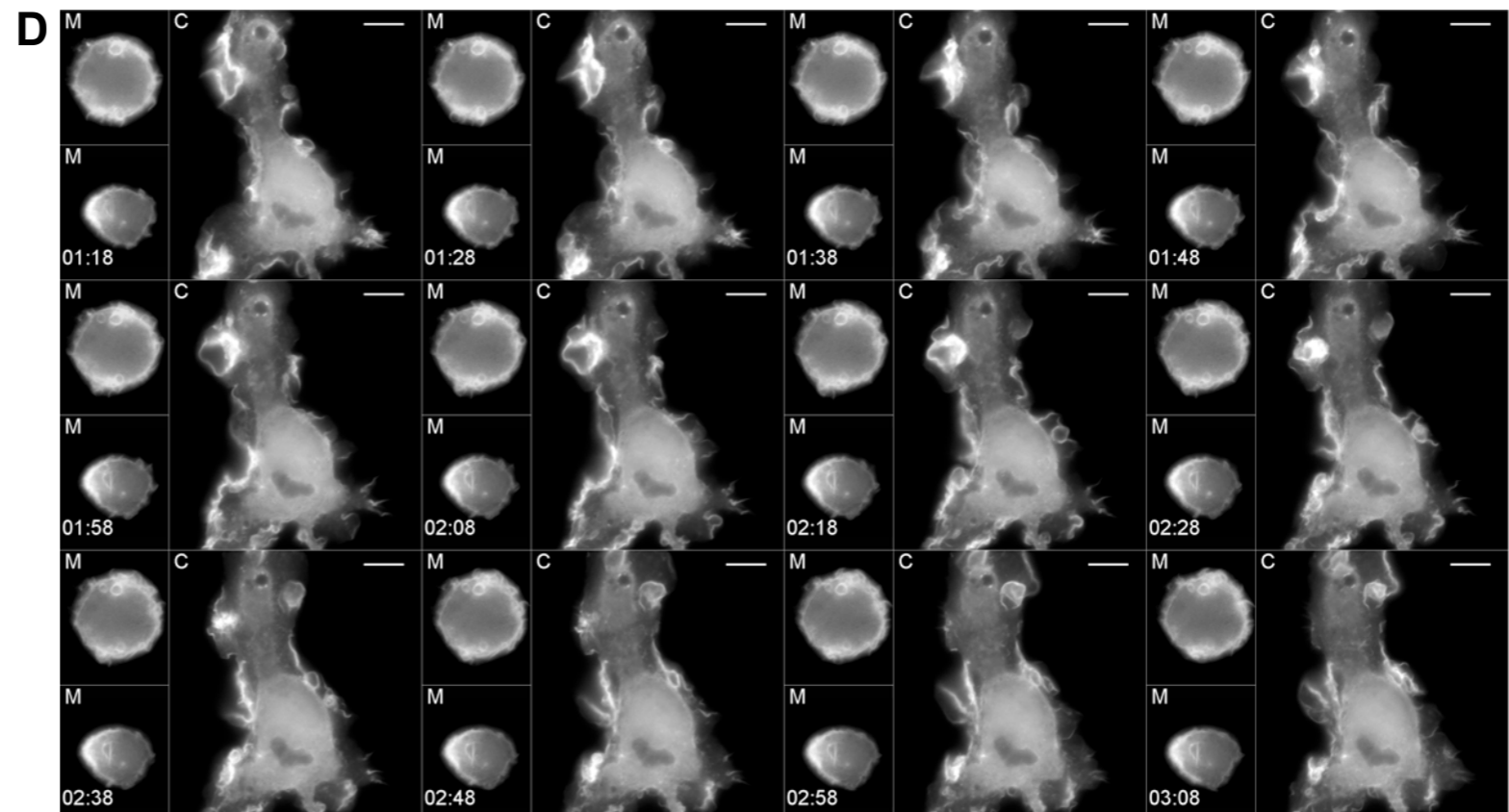

Supplement: Figure S1 — (A) Expression of n-cofilin and ADF in bone marrow and bone marrow derived cells. Recombinant GST-fusion proteins were titrated against lysate from total bone marrow (bm) and bone marrow derived macrophages after 6 days of culture (BMM). Quantitative analysis (histogram, lower panel) shows a 1∶1 ratio of n-cofilin and ADF in bm, while in BMM the ratio changes to about 8∶1; (B) Expression of macrophage surface markers on BMM after 6 days of culture. Macrophages from n-cofflx/Δ,Mx1-cre and control mice expressed comparable levels of CD86, MHC class II, and CD11b, the escaper cell population is characterized by a normal phalloidin signal; (C) Morphology of n-cofnull macrophages by scanning electron microscopy. Mutant cells showed ruffles but no leading edge, and poor cell polarity (lower panel). Control macrophages had the typical flat morphology, membrane ruffles and extended lamellipodia (upper panel). Images were taken at 5000x magnification; (D) Cytoskeletal dynamics of n-cofnull macrophages. Control (C) and n-cofnull macrophages (M) were transfected with LifeAct-GFP to visualize actin dynamics in live cells. Video sequences illustrate the high dynamics of actin rich extensions in control cells, while n-cofilin mutant macrophages show very limited actin remodelling (see video S1, S2, S3). (PDF) [file pone.0036034.s001.pdf]

Supplementary Figure 2: Jönsson et al.,

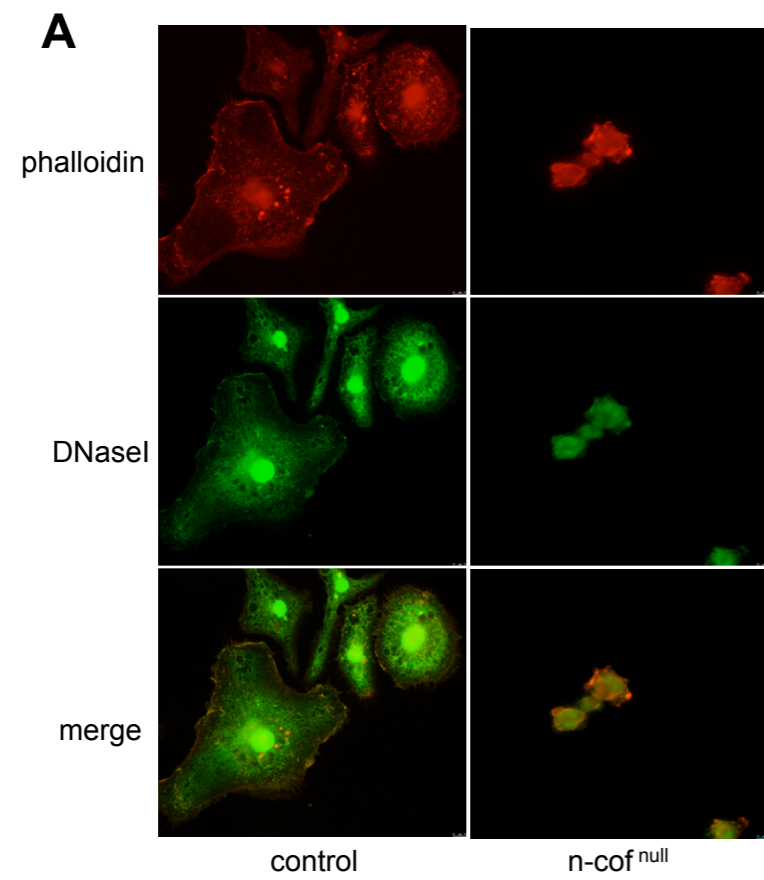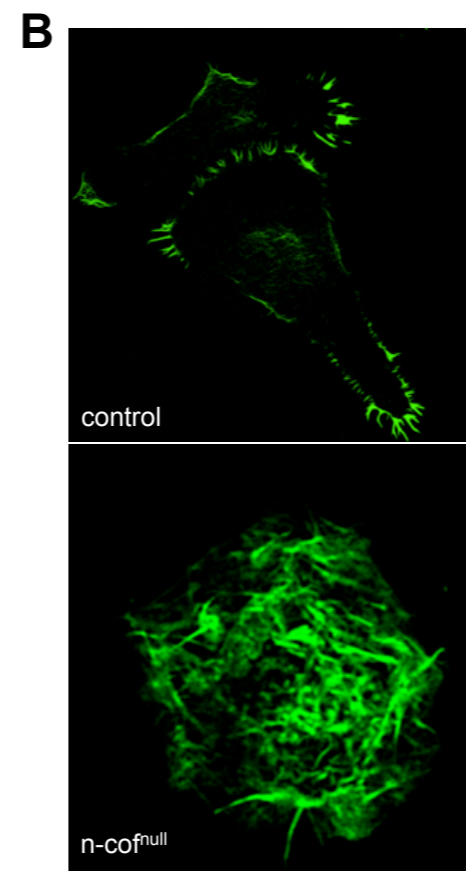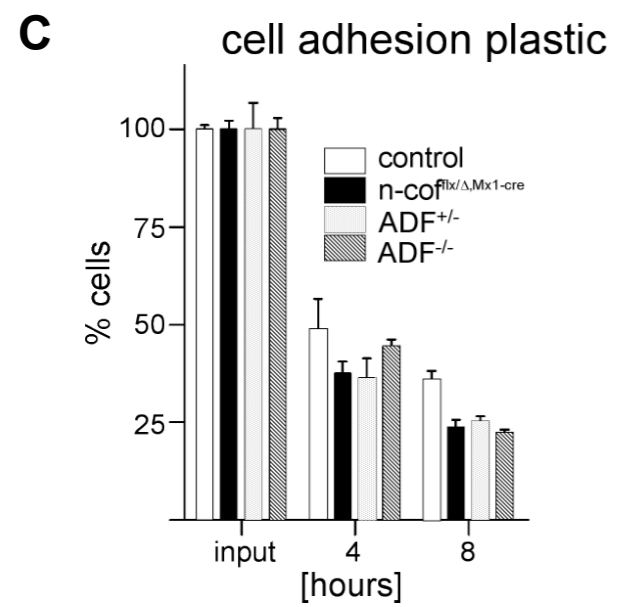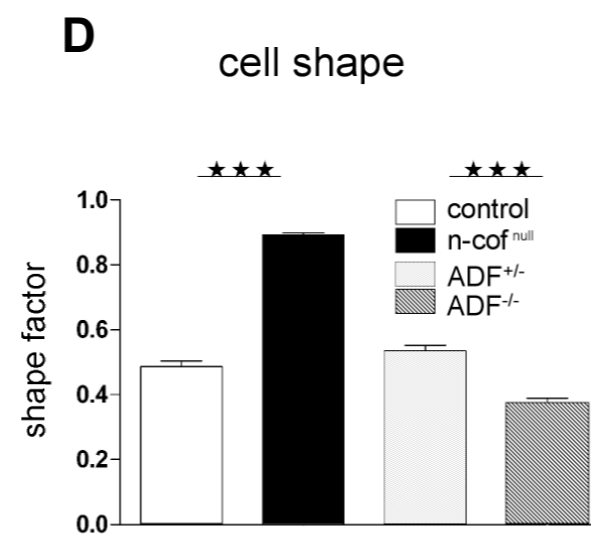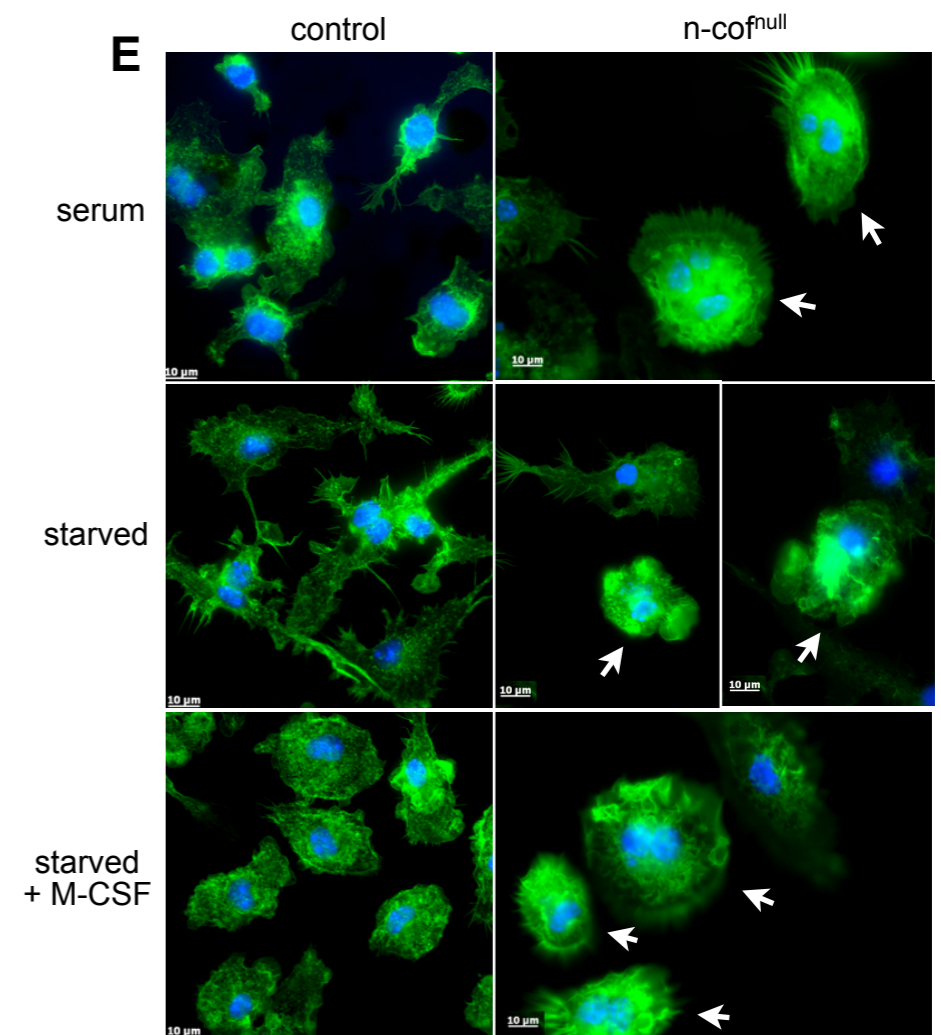

Supplement: Figure S2 — (A) Phalloidin and DNaseI staining of fixed control and n-cofilin mutant macrophages. Note that the exposure times for the phalloidin stained n-cofilin mutant cells (n-cofnull) had to be reduced to 1/5 of the controls in order to avoid overexposure. The same magnification of control and n-cofnull cells is shown; (B) ‘Actin Footprint’ structure of control and n-cofnull macrophages. Live macrophages were extracted with 0.05% saponin, after fixation in 4% PFA actin structures were stained with phalloidin (green). In control macrophages only the actin-rich contact sites were preserved, while in n-cofnull macrophages most of the cytoskeleton was resistant to extraction; (C) Adhesion of bone marrow derived cells on plastic. Bone marrow cells from mutant macrophages (n-cofilinflx/Δ,Mx1-cre), as well as ADF−/− macrophages showed normal attachment to plastic surface; (D) Cell shape as expressed by the ‘shape factor’ (SF). SF = 4ΠA/P2 (A: area, P: cell perimeter). A SF of 1 describes a perfect circle, lower values are a measure of cell extension. N-cofnull macrophages were almost round (SF = 0,89±0.01), while ADF−/− macrophages were significantly more elongated (SF = 0.38±0.01) when compared to control cells (SF = 0.49±0.02) and ADF+/− cells (SF = 0.53±0.02). ncells>100, student's test: n-cofnull:control p<0,0001; ADF−/−:ADF+/− p<0,0001; (E) Starved control and n-cofflx/Δ,Mx1-cre BMM were stimulated for 5 min with 0.5 µg/ml M-CSF. Cells were labelled with phalloidin (green)/DAPI (blue). Note that n-cofnull macrophages (white arrows) are resistant to serum deprivation, while control macrophages start retracting cell protrusions. Upon M-CSF stimulation, control BMM re-form cell extensions. Mutant BMM also responded with increased cell spreading. (PDF) [file pone.0036034.s002.pdf]

Supplementary Figure 3: Jönsson et al.

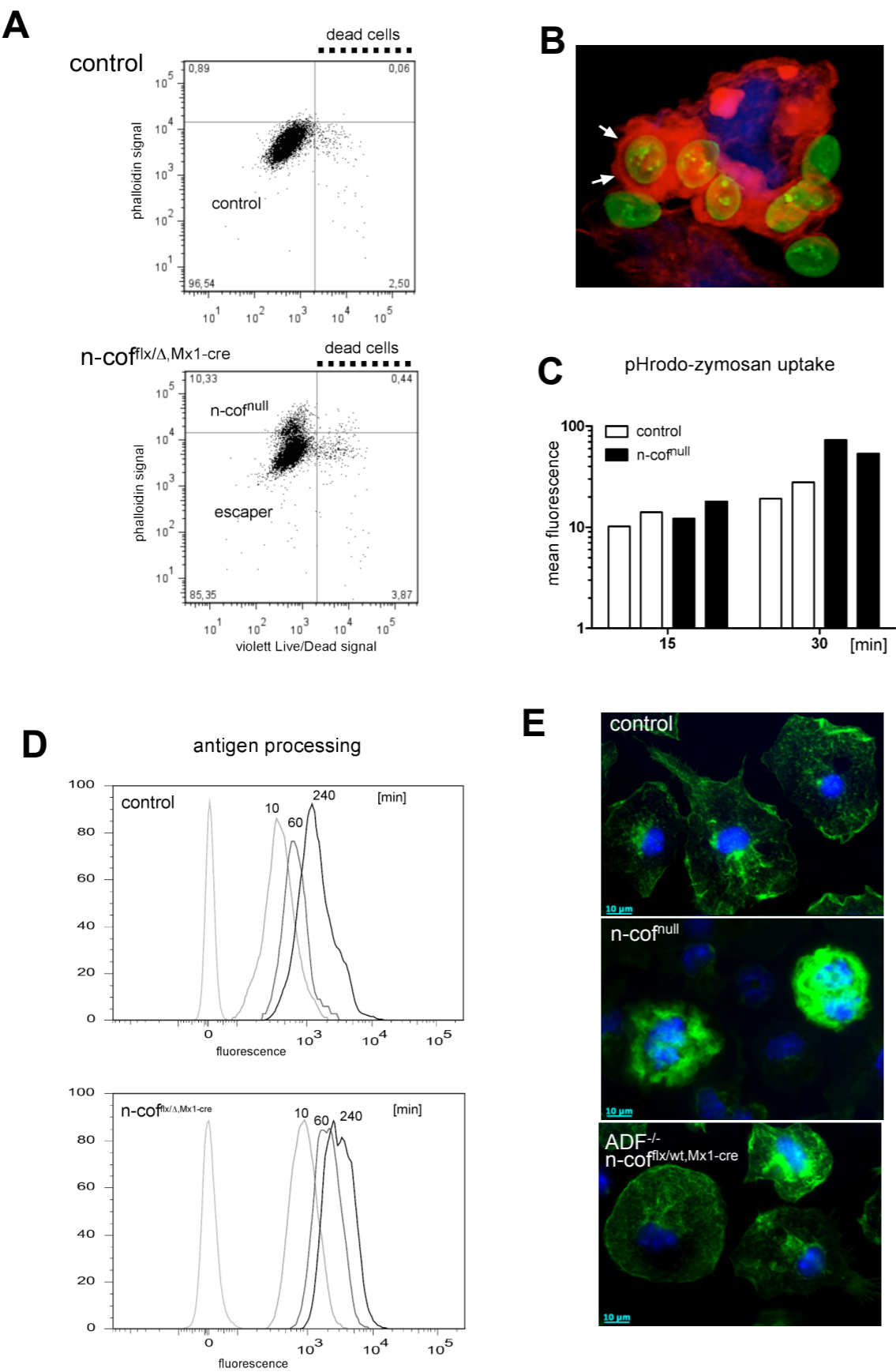

Supplement: Figure S3 — (A) Viability of control and n-cofflx/Δ,Mx1-cre macrophages, differentiated for 6 days in culture, was shown by staining with violet LIVE/DEAD (see methods). Respective percentages of cells represented in the quadrants are indicated. In the n-cofnull and escaper population more than 95% of cells were viable; (B) Phagocytosis of pHRodo-zymosan partricles. pHRodo-zymosan was offered for 15 and 30 min to control and n-cofflx/Δ,Mx1-cre macrophages. Uptake was stopped by washing cells with cold PBS. FACS analysis was used to quantify the uptake of particles into the lysosomal compartment. Two independent experiments each are shown for the control (n-cofwt/wt,Mx1-cre) and mutant macrophage cultures (n-cofflx/Δ,Mx1-cre). (C) Phagocytic cup formation occurs in n-cofnull macrophages (lower panel). After 15 minutes of FITC-labelled zymosan uptake (green), cells were fixed and counterstained for F-actin (phalloidin, red) to visualize the formation of the F-actin-rich cup (see arrows). Nuclei are shown in blue (DAPI); (D) N-cofilinflx/Δ,Mx1-cre macrophages are able to process antigens such as DQ-ovalbumin. Control and n-cofilinflx/Δ,Mx1-cre macrophages were allowed to internalize and process DQ-ovalbumin for 10, 60 and 240 minutes followed by FACS measurement of the fluorescence increase due to the generation of peptides from proteolyzed DQ-ovalbumin. Note that the kinetics of processing are slightly faster in n-cofnull macrophages; (E) Specificity of n-cofilin and ADF activity in macrophages. Complete deletion of n-cofilin rather than a general decrease of ADF/cofilin activity is responsible for the observed phenotype. The cell shape of compound mutants (ADF-/-/n-cofilinflx/wt,Mx1-cre) lacking both alleles of ADF and one allele of n-cofilin is comparable to control macrophages. (PDF) [file pone.0036034.s003.pdf]
